# Gadolinium-Labelled Cell Scaffolds to Follow-up Cell Transplantation by Magnetic Resonance Imaging

**DOI:** 10.3390/jfb10030028

**Published:** 2019-07-02

**Authors:** Valeria Catanzaro, Giuseppe Digilio, Federico Capuana, Sergio Padovan, Juan C. Cutrin, Fabio Carniato, Stefano Porta, Cristina Grange, Nenad Filipović, Magdalena Stevanović

**Affiliations:** 1 Department of Science and Technologic Innovation, Università del Piemonte Orientale “Amedeo Avogadro”, Viale T. Michel 11, I-15121 Alessandria, Italy; 2 Department of Molecular Biotechnology and Health Science & Center for Molecular Imaging, University of Turin, Via Nizza 52, 10126 Torino, Italy; 3 Institute for Biostructures and Bioimages (CNR) c/o Molecular Biotechnology Center Via Nizza 52, 10126 Torino, Italy; 4 Department of Medical Sciences, University of Turin, Via Nizza 52, 10126 Torino, Italy; 5 Institute of Technical Sciences of the Serbian Academy of Sciences and Arts, Knez Mihailova 35/IV, 11000 Belgrade, Serbia

**Keywords:** cell scaffold, graft transplantation, gadolinium, Magnetic Resonance Imaging, biomaterial, human mesenchymal stromal cells (hMSC), immune response

## Abstract

Cell scaffolds are often used in cell transplantation as they provide a solid structural support to implanted cells and can be bioengineered to mimic the native extracellular matrix. Gadolinium fluoride nanoparticles (Gd-NPs) as a contrast agent for Magnetic Resonance Imaging (MRI) were incorporated into poly(lactide-co-glycolide)/chitosan scaffolds to obtain Imaging Labelled Cell Scaffolds (ILCSs), having the shape of hollow spherical/ellipsoidal particles (200–600 μm diameter and 50–80 μm shell thickness). While Gd-NPs incorporated into microparticles do not provide any contrast enhancement in T_1_-weighted (T_1_w) MR images, ILCSs can release Gd-NPs in a controlled manner, thus activating MRI contrast. ILCSs seeded with human mesenchymal stromal cells (hMSCs) were xenografted subcutaneously into either immunocompromised and immunocompetent mice without any immunosuppressant treatments, and the transplants were followed-up in vivo by MRI for 18 days. Immunocompromised mice showed a progressive activation of MRI contrast within the implants due to the release of Gd-NPs in the extracellular matrix. Instead, immunocompetent mice showed poor activation of MRI contrast due to the encapsulation of ILCSs within fibrotic capsules and to the scavenging of released Gd-NPs by phagocytic cells. In conclusion, the MRI follow-up of cell xenografts can report the host cell response to the xenograft. However, it does not strictly report on the viability of transplanted hMSCs.

## 1. Introduction

Despite the initial great promise of cell therapy, clinical trials have come up in many cases with conflicting results and controversial clinical outcomes [1,2,3]. Known issues for the success of the treatment are linked to the delivery of a stable and viable population of stem cells at the damaged site, that is in turn strongly dependent on the administration route and dose. The abrupt change of microenvironment conditions after grafting cells expanded in vitro into the host may further contribute to cell loss. Finally, the host immune response is often indicated as a major cause of graft rejection.

Several strategies have been proposed to improve the delivery and retention of a stable and dense population of therapeutic cells at the damaged site. Amongst these, the main strategy relies upon either cell (micro)encapsulation and/or cell scaffolding [4,5,6,7]. Cell encapsulation makes use of biocompatible hydrogels (e.g., alginate, hyaluronic acid, gelatin/collagen) to create a 3D environment in which cells can be embedded. Cell encapsulation is often intended to shield therapeutic cells from the host hostile environment by preventing the infiltration of immune cells. As an example, embedding of human mesenchymal stromal cells (hMSCs) in a gelatin matrix prior to surgical grafting into the myocardium in swine proved to be critical for increasing intra-myocardial retention of hMSCs [8]. Cell scaffolds are made by synthetic biocompatible materials such as poly(lactide-co-glycolide), PLGA, bioengineered to provide a solid structural support to implanted cells and to mimic the extracellular matrix, allowing cells to proliferate and/or differentiate in the desired way [9]. Seeding neuronal stem cells on solid supports (i.e., the cell scaffolds) prior to implantation has been shown to be essential to elicit tissue regeneration in brain repair after stroke [6]. Solid scaffolds can be fabricated in different size and forms (including solid foams, nanofibers, microspheres, or microtubules), depending on their specific application [10]. Very interestingly, scaffolds can be designed to release bioactive molecules, such as growth factors, DNA, or drugs, in a controlled manner to facilitate tissue regeneration [11].

The variability among individuals in regards to graft immune rejection still remains an issue [12]. Immune response can be directed against cells, against their solid supports or encapsulating materials, triggered by the surgical act, or a combination of them. Foreign Body Response (FBR) has been shown to cause fibrosis through activation of macrophages and dendritic cells as a reaction to alginate capsules [13]. In the absence of methods enabling the real time-monitoring of the integrity of the cell population and of their function, it is therefore very hard to assess whether inconsistent endpoint clinical outcomes are due to graft rejection or poor intrinsic efficacy of the therapy. To bridge the knowledge gap between cell therapy administration and endpoint health outcomes, there is a need for non-invasive, clinically compliant methods enabling to follow-up longitudinally the cell state [14]. Magnetic Resonance Imaging (MRI) has gained a prominent role in the field of cell therapy because it is minimally invasive, can image tissues at any depth in the body, and offer an acceptable compromise between time/space resolution and sensitivity. Cell tracking by MRI allows one to follow the migration and homing of cells after introduction within the host body [14,15]. An essential requirement to track stem cells by MRI is the incorporation of an imaging probe (also called the contrast agent, CA) into the cells of interest in order to distinguish them from their surroundings (direct labelling). Superparamagnetic Iron Oxide (SPIO) nanoparticles have been extensively used to track cells by MRI. Graft rejection can be assessed by following the homing of intravenously administered SPIO-labeled macrophages in kidney transplantation [16] and xenotransplantation models [17]. Impairment of SPIO-labelled pancreatic islet allografts due to rejection can be predicted in advance of functional failure by MRI [18]. There are, however, drawbacks with the use of SPIO-labelled cells linked to the long-term persistence of SPIO signal after the death of transplanted cells, leading to false results [19,20]. Furthermore, there are reports on the detrimental effects on stem cell multipotentiality linked to the high payload of intracellular labels [21].

As an alternative to direct cell labelling, labelling of the extracellular matrix within cell capsules was proposed, under the assumption that the cell state can be inferred by assessing the capacity of the extracellular matrix (ECM) microenvironment to support cell survival [22,23]. Chan et al. reported a pH-nanosensor-based MRI probe that could monitor microenvironment pH within cell-loaded microcapsules in vivo and non-invasively. Onset of acidosis was shown to report about cell death within grafted capsules [24]. In another study, cell infiltration and FBR response to alginate capsules could be detected based on the Magnetization Transfer MR properties of the ECM microenvironment [13]. We recently presented silica microspheres decorated with Gd-complexes as a redox-responsive MRI probe to follow-up hypoxia within cell-embedding hydrogels [25]. In principle, a variety of microenvironment features can be probed, providing that a suitable responsive system is available (for instance, liposomes grafted with MRI-labelled substrates for matrix metalloproteinases can be used to label the extracellular microenvironment for probing matrix remodeling) [26].

**Scheme 1:**
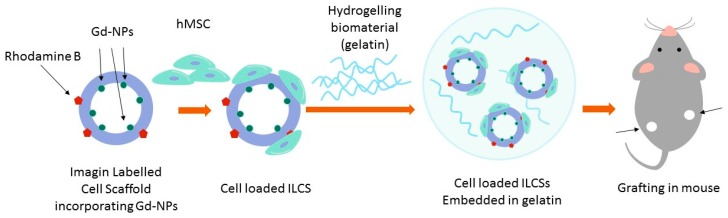
Design of Imaging labeled Cell Scaffolds (ILCSs) for the follow-up of cell therapy. ILCSs are first seeded with hMSCs, then grafted subcutaneously into mice in association with a gelatin matrix.

**Scheme 2:**
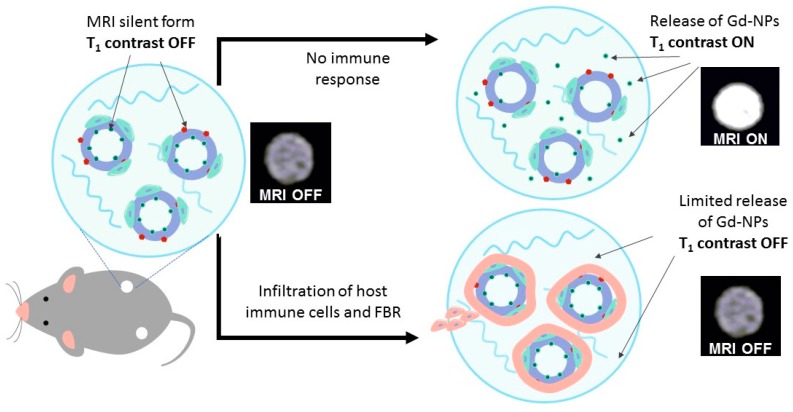
Principle of the responsivity of the ILCS based system to host cell infiltration.

In this study, we present novel biocompatible microparticles integrating a cell scaffolding function for hMSCs with an imaging function to monitor immune response by MRI. Such Imaging Labelled Cell Scaffolds (ILCSs) are designed to be seeded with cells, then embedded into a gelatin matrix, and finally grafted into the host (Scheme 1). ILCSs are based on microparticles composed of poly(lactide-co-glycolide) (PLGA) and chitosan (CHT). These microparticles incorporate gadolinium fluoride nanoparticles (Gd-NPs) as the T_1_ MRI contrast agent [27]. As such, the incorporated Gd-NPs are MRI silent, as they are shielded from any interaction with bulk water molecules. However, Gd-NPs are slowly released into the extracellular milieu, where they become activated for T_1_-dependent MRI contrast enhancement (Scheme 2). Due to their size (hydrodynamic diameter of ca. 12 nm; inorganic core 2 nm) [27] and interactions with the macromolecular components of the extracellular matrix, diffusion of Gd-NPs within the hydrogel is restricted, and local activation of MRI contrast is expected. Foreign body response against scaffolded cells is expected to lead to fibrosis, encapsulation of ILCSs, and scavenging of released Gd-NPs by phagocytic cells, preventing ultimately the activation contrast enhancement.

## 2. Results

### 2.1. Synthesis and Characterization of ILCSs

**Figure 1:**
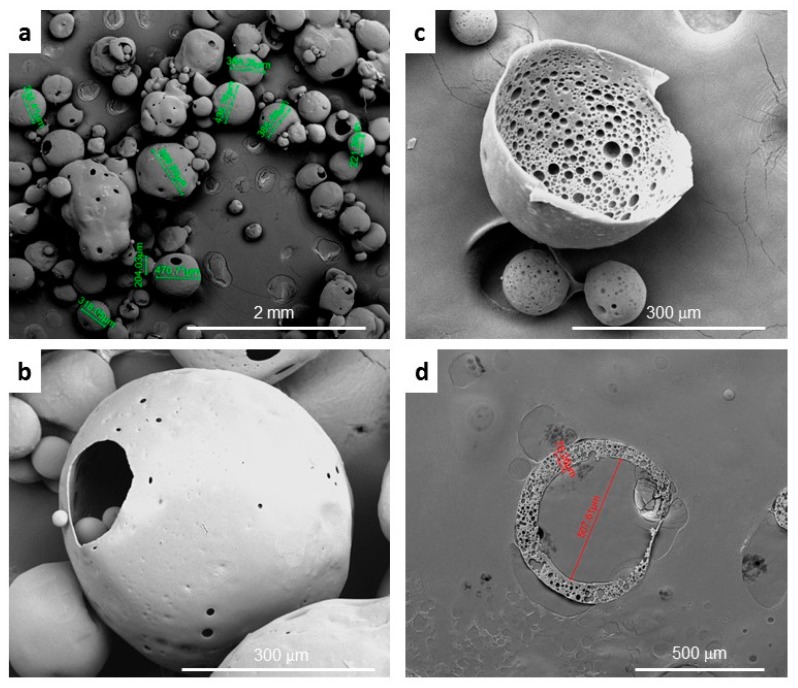
SEM micrographs of ILCSs. (**a**) Microparticles are hollow and quite poly-dispersed in size, ranging from 200 μm to 600 μm. (**b**) Large and tiny holes on the surface reveal the inner cavity of the microspheres (**c**,**d**). Ten micron thick slices of the microparticles show a sponge like structure within the particle shell.

Microparticles were synthesized by the modified emulsion/solvent evaporation technique that is commonly used for encapsulation of hydrophilic components, such as the suspension of Gd-NPs, into the hydrophobic PLGA polymer. The main steps in the synthesis were: (i) Preparation of primary emulsion (*w*/*o*) by emulsifying the aqueous suspension of Gd-NPs with PLGA dissolved in chloroform; (ii) preparation of a second emulsion by transferring the primary emulsion into an excess volume of water containing a mixture of CHT and poly(vinyl alcohol) (PVA) under vigorous stirring, and (iii) final removal of the solvent accomplished by evaporation or extraction. The ILCSs synthetized by such procedure have a payload of Gd-NPs of 18 ± 2 nmol_Gd_ per milligram of ILCS as far as evaluated by means of Inductively Coupled Plasma-Mass Spectrometry (ICP-MS). Scanning Electron Microscopy (SEM) images show a poly-dispersion of the particles, roughly splitting into a population of spherical particles having a diameter less than 100 μm and a population of larger, hollow particles (Figure 1).

**Figure 2:**
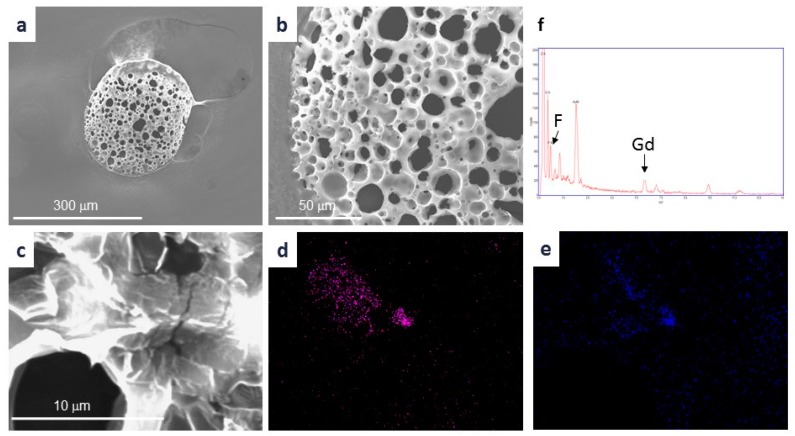
Energy Dispersive X-ray Spectroscopy (EDS) of ILCSs sections. (**a**) SEM image 500×; (**b**) SEM image 2000×; (**c**) SEM image 6000×; (**d**) EDS gadolinium map; (**e**) EDS fluorine map; (**f**) EDS spectrum with the indication of gadolinium and fluorine K-lines.

Although most of the larger particles have an almost spherical shape with a diameter in the range of 200 μm to 600 μm, others are ellipsoidal or cup-shaped. All large particles present several holes on their surface that in some instances reveal the inner cavity. When cut into 10 μm thick slices, hollow particles yield ring-shaped sections, whose thickness corresponds to that of the particle shell (typically in the range 50–80 μm). The ring-shaped sections reveal a sponge-like structure, characterized by a high density of spherical cavities likely left over by the evaporating solvent. Gadolinium analysis by Energy Dispersive X-ray Spectroscopy (EDS) done on ring sections shows hot spots of gadolinium atoms within the porous structures (Figure 2). Fluorine is co-localized with gadolinium, indicating that Gd-NPs did not decompose during particle formation. Gd-NPs were detected within the polymer matrix by Transmission Electron Microscopy (TEM, Figure S1).

### 2.2. Release of Gd-NPs In Vitro

**Figure 3:**
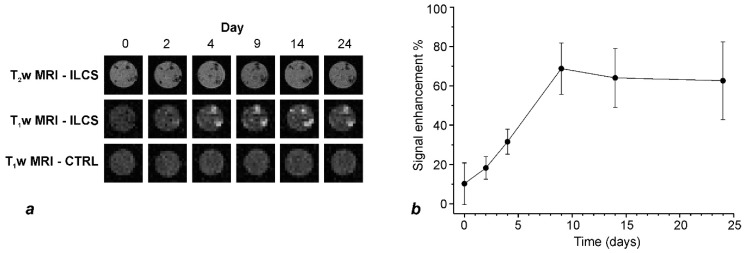
MRI of agar phantoms embedding ILCSs to monitor the activation of contrast enhancement over time. (**a**) T_2_w MRI of ILCS (top row), T_1_w MRI of ILCS (middle row) showing the time dependent activation of MRI contrast, and T_1_w MRI of particle-free agar (ctrl, bottom row); (**b**) Signal enhancement measured from T_1_w-MR images as a function of the incubation time (days). Error bars represent ±SD from triplicate.

To assess Gd-NPs’ release from ILCSs, microparticles were embedded into 1% agar and agar phantoms were incubated at RT for up to 24 days, while the MRI Signal Enhancement (SE) was monitored at regular time intervals by means of T_1_ weighted (T_1_w) and T_2_ weighted (T_2_w) MRI. ILCSs were visible by T_2_w-MRI as hypointense signal all along the follow-up time (Figure 3a). No gadolinium-dependent signal enhancement was detected at day-0 in T_1_w MR images, and ILCSs could be barely distinguished from the agar embedding them. However, ILCSs progressively developed gadolinium dependent contrast, appearing as bright spots in T_1_w images. This indicated that an appreciable fraction of Gd-NPs trapped within the PLGA polymer were released into the agar phase. Figure 3b shows that the SE increases up to a plateau as a function of the incubation time.

### 2.3. Biocompatibility

**Figure 4:**
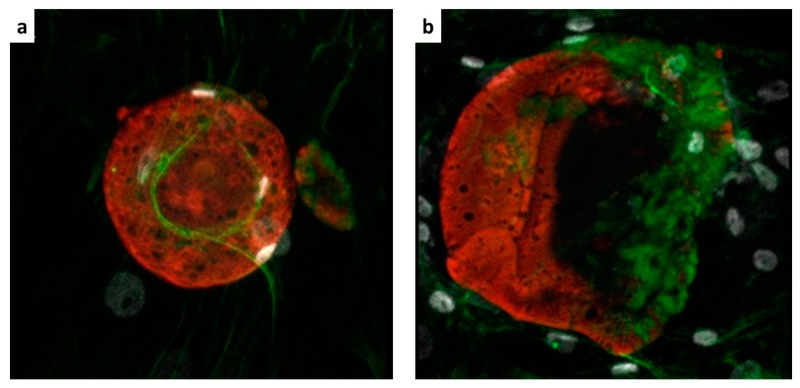
Confocal images of hMSCs seeded on ILCSs after (**a**) one and (**b**) two weeks of culture. ILCSs are shown in red (fluorescent labelling with rhodamine B); The cytoskeleton is visible in green (phalloidin-FITC labelling); Cell nuclei are shown in white (Hoechst staining).

The biocompatibility of ILCSs has been assessed by incubating them with hMSCs for up to 20 days, and by evaluating cell viability and morphology at regular time intervals. To this purpose, hMSCs were cultured on a plate with ILCSs (either containing or not Gd-NPs). Confocal microscopy images taken after one and two weeks of incubation showed that ILCSs were easily colonized by hMSCs, with cells adhering to the microsphere surface or in between two adjacent microspheres (Figure 4; optical images as well as SEM images of hMSCs adhering to the scaffolds are shown in Figures S2 and S3). Cells were well-spread on particles and able to form large clusters, indicating a normal morphology. The cell-seeded scaffolds were taken out the plate and placed into a new culture plate. Colonization of the plate with hMSCs derived from ILCSs was achieved after one week, as expected for cells behaving normally. No adverse effect on cell proliferation or morphology due to the ILCSs, either containing Gd-NPs or not, were detected. hMSC viability in the presence of ILCSs (either containing or not Gd-NPs) was comparable to the one of hMSC cultured in plate, as far as evaluated by the MTT assay at different time points. Flow cytometry analysis after incubation with ILCSs revealed the positive expression of the classical mesenchymal stem cell markers such as CD105, CD73, CD90, CD146 and CD44 (Figure S4a). The percentage of markers expression was comparable to the one of hMSCs cultured in the absence of ILCSs. hMSCs cultured for three weeks in adipogenic differentiation were able to undergo adipogenic differentiation (Figure S4b) with positive stain for lipid droplets. Moreover, cells maintained in osteogenic differentiation medium exhibited deposits of calcium, indicating an osteogenic differentiation (Figure S4b). All these findings pointed out a very good biocompatibility of ILCSs with no detrimental effects on multipotentiality.

### 2.4. MRI Follow-up In Vivo

**Figure 5:**
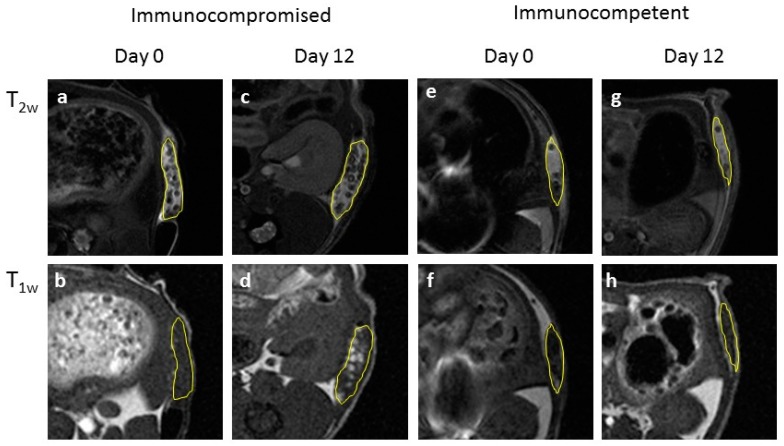
Expansions of MR images around the +hMSCs grafts (ROI marked in yellow) in an immunocompromised NSG mouse (**a**–**d**) and an immunocompetent FVB mouse (**e**–**h**). ILCSs seeded with hMSCs appear as hypointense spots on the uniform matrigel background in T_2_w-MR images, both for the NSG (**a**,**c**) and the WT mouse (**e**,**g**). In the T_1_w-MR image at Day 0, ILCSs can be hardly distinguished from the background both in the NSG (**b**) and the FVB (**f**) mouse. After 12 days, ILCSs are clearly bright in the T_1_w-MR image of the NSG mouse (**d**) but not in that of the FVB mouse (**h**).

**Figure 6:**
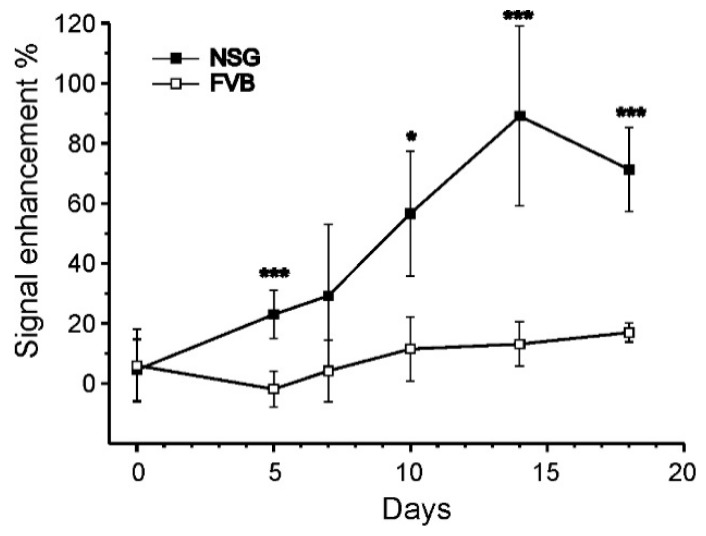
Time course of T_1_w MRI signal enhancement (SE) of ILCSs grafted subcutaneously in mice. The SE increases with time in immunocompromised (NSG) mice (solid squares), while it does not change appreciably in immunocompetent (FVB) mice. The SE was averaged over *n* = 4 implants for NSG mice and *n* = 4 implants for FVB mice (error bars are ±SD; t-test: * *p* < 0.05, *** *p* < 0.005).

Matrigel-embedded, scaffolded cells were implanted subcutaneously into either immunocompetent (FVB) or immunocompromised (NSG) mice. Immunocompromised mice were chosen to mimic an ideal case with minimal immune response. Immunocompetent mice were expected to elicit immune response, as they received cell xenografts through a surgical act without any immunosuppressant treatment. Each mouse received two implants, one in the left flank containing scaffolded hMSCs (+hMSC), and one in the contralateral side containing the scaffold only (−hMSC). MR images showing the +hMSC implant of an immunocompromised mouse acquired at day-0 (i.e., the day after surgery) and at Day 12 p.i. are shown in Figure 5. The cell loaded ILCSs appeared as hypointense spots over the homogenous gelatin background in T_2_w MR images all along the follow-up period. The size of such spots suggested some extent of scaffold aggregation. In the T_1_w-MR image at Day 0, both ILCSs and the gelatin background had a very low signal intensity, with ILCS being indistinguishable from the background. After 12 days, ILCSs lighted up to hyperintense signal, indicating the release of Gd-NPs from the scaffold shell. The same behaviour was observed also for the −hMSCs contralateral implant (Figure S5). The MRI follow-up protocol was applied to the immunocompetent (FVB) mice as well. The baseline MR images (day-0) of +hMSC implants were very similar to those of the immunocompromised mouse, with ILSCs appearing as hypointense spots in T_2_w MR images and being undistinguishable from the background in T_1_w MR images. However, no or poor activation of gadolinium dependent contrast was observed all throughout the follow-up period (18 days). There was no activation of gadolinium dependent contrast in the –hMSC implants as well. Figure 6 shows the time course of activation of gadolinium-dependent MR contrast enhancement for all implants considered (*n* = 8). Immunocompromised mice showed activation of T_1_ contrast in all their implants (*n* = 4), while poor activation was observed for immunocompetent mice (*n* = 4).

### 2.5. Histology of Excised Cell Grafts

**Figure 7:**
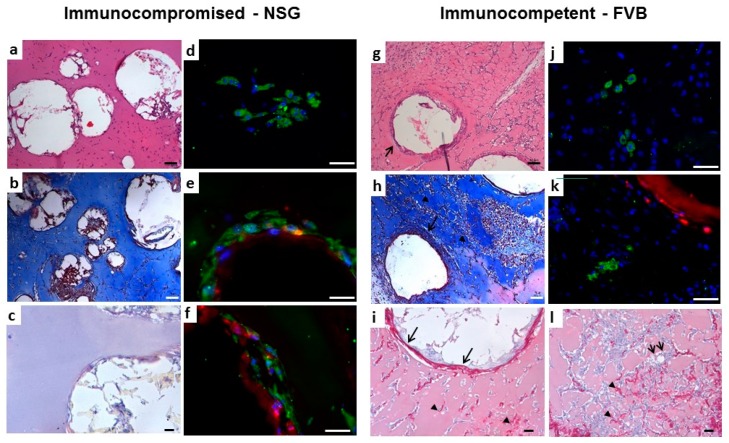
Histology of +hMSC subcutaneous cell implants excised from a representative immunocompromised NSG mouse (**a**–**f**) and immunocompetent FVB mouse (**g**–**l**). (**a**–**c**) H&E, Masson and Sirius red stains of NSG implants; (**d**–**f**) HLA specific immunostaining of NSG implants (HLA positive cells are green; cell nuclei are blue; the ILCS surface is red, scale bar 25 μm); (**g**–**i**,**l**) H&E, Masson and Sirius red stains of FVB implants. Arrows indicate microspheres delimited by an intense fibrotic reaction. Arrow heads point to the vascular organization of the matrigel. Double arrows indicate macrophage foamy cells; (**j**,**k**) HLA specific immunostaining of NSG implants (color coding as in (**d**–**f**); scale bar 25 μm). Scale bar: 50 μm for (**a**,**b**,**g**,**h**); 25 μm for all others.

On Day 20, mice were sacrificed and the implants were recovered (a representative one is shown in Figure S6) and histological analysis was performed by hematoxylin/eosin (H&E), Masson trichrome, and Sirius red stains to assess the extent of host cell infiltration and matrigel reorganization. Immunocompromised NSG mice showed very low infiltration of host cells within the matrigel matrix, no vascular organization or collagen remodelling of the matrigel, as seen by the uniform stain of the matrigel (Figure 7). Spare foamy cells were detected within the matrigel. There were no significant differences in the histologic appearance between implants seeded (+hMSC) or not (−hMSC) with cells (the latter is shown in Figure S7). A large number of hMSC transplanted into mouse were found to be still adhering to the scaffold by immunostaining specific for the HLA antigen (Figure 7e,f). These cells appeared spread, forming clusters and dividing, indicating a healthy condition. In addition, some clusters of hMSCs were also found within the matrigel phase, indicating that hMSCs have also migrated and proliferated (Figure 7d).

In the immunocompetent FVB mice, the hydrogel matrix was highly infiltrated with cells as shown by the H&E, Masson trichrome, and Sirius red stains of both +hMSC and –hMSC implants. Most of the microspheres were delimited by an intense fibrotic reaction, and extensive vascular reorganization of the matrigel took place. Several foamy cells were detected, indicating phagocytosis of the degraded scaffolds. Notwithstanding severe infiltration of host cells, the HLA immunostaining revealed that hMSCs with normal morphology were still present in the implant, mostly residing in the hydrogel matrix rather than on the scaffold surface (Figure 7j,k). Apparently, migration of transplanted cells from the scaffold to the hydrogel bulk took place.

## 3. Discussion

The delivery of therapeutic cells in association with biomaterials provides the outstanding opportunity to add microenvironment-responsive imaging labels in a form interspersed with therapeutic cells. While the incorporation of MRI probes into the extracellular space within hydrogel-based microcapsules has several examples [22], the labelling of cell scaffolds received comparatively much less attention [28,29]. To the best of our knowledge, this is the first study with cell scaffolds labelled with MRI probes endowed with responsivity to the cell extracellular microenvironment. The system we designed is intended to detect the infiltration of host cells in the gelatin matrix, embedding scaffolded cells. It is worth emphasizing that the matrigel we used for cell embedding does not provide immune-isolation in itself. As opposite, it is permeable to host cells and allows for matrix remodelling. It was chosen for a better delivery of cell grafts, and to model cell infiltration within the graft. If immunoisolation of embedded cells is sought for a specific implementation of cell therapy, microencapsulation through suitable hydrogelling polymers, such as alginate, must be used instead.

We performed an initial evaluation of our follow-up system on either immunocompromised or immunocompetent mice, under the hypothesis of no immune response in the former animals, and occurrence of immune response in the latter. Endpoint ex vivo histology confirmed such an hypothesis, as we observed poor or no infiltration of host cells with immunocompromised mice, and extensive infiltration and matrix remodelling in immunocompetent mice. The extent of cell infiltration was not affected by the presence of hMSCs, as no significant histologic differences between +hMSCs and –hMSCs cell grafts were found. The endpoint histologic observation was nicely paralleled by MRI observations. We detected a progressive appearance of MRI contrast in grafts devoid of host infiltrate. On the other hand, poor MRI contrast appeared in the presence of appreciable infiltration, likely due to the encapsulation of ILCSs within fibrotic capsules and to the scavenging of released Gd-NPs by phagocytic cells. Therefore, the imaging response well correlates with cell infiltration within the matrigel phase.

The relationship between the MRI response and cell survival is less clear. Surviving hMSCs were detected ex vivo both in infiltrated and non-infiltrated grafts. The survival of xenogeneic cells up to 20-days post transplantation even under the circumstance of strong infiltration can be due to the type of cells we have used in our work. A number of studies have shown that hMSCs have unique immunoregulatory functions [30,31,32] that make them promising for long-term cell therapy. Human MSCs were xenografted in rat and swine without immunosuppressant treatment and gene expression was imaged by PET [8]. Human MSCs were xenotransplanted in intervertebral discs in a xenogeneic porcine model and shown to survive for months [33]. Mouse MSCs showed unique immunologic tolerance, allowing their engraftment into a xenogeneic environment [34]. As +hMSC implants were indistinguishable from –hMSC implants, it can be speculated that immune response is directed against the microparticles rather than against xenogeneic cells. This would be in line with other reports, showing a mild FBR directed against alginate microcapsules [13]. In summary, while MR image response is clearly related to host cell infiltration, it cannot be unambiguously related to the death of transplanted cells. Further quantitative assessment of survival of hMSCs cells within in vivo xenograft is needed to shed light on this matter.

## 4. Materials and Methods

### 4.1. Synthesis and Characterization of ILCSs

Citrate-coated GdF_3_ nanoparticles (Gd-NPs) were prepared as described previously [27]. ILCSs incorporating Gd-NPs were prepared by a modified double emulsion-solvent evaporation technique [35]. Firstly, one gram of commercial PLGA granules (Durect Lactel Absorbable Polymers, Cupertino, CA, USA; lactide-to-glycolide ratio 50:50; Mw 40,000–50,000 g/mol) was dissolved into 5 mL of chloroform for 24 h (solution A). Solution B was prepared by dissolving 100 mg of PVA (Sigma-Aldrich, St. Louis, MO, USA, Mowiol™ 4–88, Mw ~31,000) in 100 mL of dd-H_2_O on a magnetic stirrer at 1200 rpm and 90 °C for 30 min. After that, solution B was left to cool down at room temperature. Meanwhile, for the preparation of solution C, 50 mg of CHT (Sigma-Aldrich, medium molecular weight, 75–85% de-acetylated) were dissolved in 50 mL of 1% (*v*/*v*) acetic acid by stirring for 30 min at 500 rpm. The pH of solution C was adjusted to pH 5 with NaOH, and solution C was added to solution B under stirring at 1200 rpm for 60 min (solution D). The primary emulsion was prepared by dropwise addition of 1 mL Gd-NPs suspension (20 mM gadolinium concentration) into solution A, under 5 min homogenization using a high-energy homogenizer (Ultra-Turrax T25, IKA, Königswinter, Germany). This PLGA/Gd-NPs emulsion was added dropwise to the mixture of CHT and PVA (i.e., solution D), under stirring at 1200 rpm, which led to the formation of a double emulsion. The final emulsion was left under stirring for 20 h to promote chloroform evaporation. The microparticles formed within the final stage were recovered by filtration and washed 3 times with dd-H_2_O on filter paper and finally allowed to dry in air at RT. Control microparticles (i.e., gadolinium-free) were prepared by the same protocol but in the absence of Gd-NPs.

The microparticles were labelled with rhodamine B to allow for optical imaging. One hundred mg of microspheres were placed in a tube with 2.5 mL of HEPES buffer (3.8 mM HEPES, 150 mM NaCl, pH 7.4). 1.2 mg of rhodamine B isothiocyanate (Sigma-Aldrich) were dissolved in 1 mL of HEPES buffer. 500 μL of the fluorescent tag solution were added to the microspheres and the reaction mixture kept under shaking at room temperature for 24 h. 3.5 mL of HEPES buffer were added to the solution and the microparticles were washed 3 times for 15 min. The micropaticles were dialyzed for 48 h against HEPES. The recovered microspheres were finally recovered by filtration and allowed to dry in air.

The gadolinium content of microparticles was determined by means Inductively Coupled Plasma-Mass Spectrometry, ICP-MS (Spectro Genesis ICP-OES, Spectro Analytical Instruments, Kleve, Germany) equipped with a crossflow nebulizer. Plasma was generated by argon with a power of 1400 W. Flow conditions, coolant flow 12.00 L/min, auxiliary flow 0.60 L/min, and nebulizer flow 1.00 L/min. Determinations were done in triplicate. Prior to the analysis, the sample was mineralized in HNO_3_/HCl 1:3 *v*/*v* (aqua regia; 5 mL) and HF (2 mL) at 100 °C for 24 h.

Scanning Electron Microscopy (SEM) micrographs were taken with a Leo 1550 SEM instrument (Zeiss, Oberkochen, Germany). Samples were mounted on aluminum stubs using a double-sided adhesive carbontape and sputtered with Au/Pd with a plasma current of 30 mA for 30 s. The thickness of Au/Pd layer was approximately 10 nm.

Optical images were acquired using an Olympus BX41 optical microscope (Olympus Corporation, Tokyo, Japan).

### 4.2. Cell Culture and Biocompatibility Assessment

Human MSCs were purchased from Lonza, cultured and characterized, as previously described [36,37,38]. hMSCs were maintained in the presence of cysteine free Mesenchymal Stem Cells Basal Medium (MSCBM, Lonza, Basel, Switzerland). All cell preparations at different passages of culture expressed the typical MSC markers, CD105, CD73, CD44, CD90, and CD146 and not hematopoietic markers like CD45, CD14 and CD34, as evaluated by flow cytometry assay [39] (see Table S1 for the complete list of antibodies used in this study).

#### 4.2.1. hMSC Culture on ILCSs

The culture of hMSCs on ILCSs was performed by incubating six mg of ILCSs (containing approx. 110 nmol of gadolinium under the form of Gd-NPs) with 1.5 × 10^4^ hMSCs in the normal culture medium. hMSCs and ILCSs were maintained in incubator at 37 °C for 4 days; after that, further 1.5 × 10^4^ hMSCs were added to the flasks on the ILCSs and maintained in culture for up to 20 days.

#### 4.2.2. Biocompatibility Assessment

The biocompatibility of the microspheres, incubated with hMSCs, has been assessed for up to 20 days by evaluating cell viability, morphology, flow cytometry analysis, and in vitro osteogenic and adipogenic differentiation.

*MTT assay.* For the evaluation of hMSCs vitality on ILCSs, the MTT (3-(4,5-dimethylthiazol-2-yl)-2,5-diphenyltetrazolium bromide) assay was performed. Briefly, ILCSs after 7 days of culture were re-suspended in 300 μL of PBS solution for efficient disaggregation. ILCS-derived cells were plated in 96-well plates in triplicates and analyzed according to the manufacturer’s instructions (Merck-Millipore, Burlington, MA, USA).

*Flow cytometry assay*. hMSCs were detached from ILCSs with non-enzymatic cell dissociation solution (Sigma, St. Louis, MO, USA), washed in PBS and then incubated for 30 min at 4 °C with the appropriate fluorescein isothiocyanate (FITC) or phycoerythrin (PE) conjugate Abs or with the irrelevant control in PBS containing 0.1 BSA (Sigma). The following antibodies, all PE- or FITC-conjugated, were used, anti-CD146, -CD105, -CD90, CD73, CD44, alpha 5 integrin. Cells were analysed on a FACS (Becton Dickinson, Franklin Lakes, NJ, USA). Ten thousand cells were analysed in each experimental point.

*In Vitro Osteogenic and Adipogenic differentiation*. For adipogenic differentiation, hMSCs were recovered after incubation with ILCSs and incubated in appropriated adipogenic differentiation medium (Lonza) [39]. The medium was changed two times per week for 3 weeks. The cells were fixed with 10% formalin for 20 min at room temperature and stained with 0.5% Oil Red O (Sigma) in methanol for 20 min at room temperature. For osteogenic differentiation, hMSCs were incubated in osteogenic differentiation medium (Lonza) [39]. The media was changed two times per week for 3 weeks. Then cells were fixed with 10% formalin for 20 min at room temperature and stained with Alizarin Red, pH 4.1 (Sigma) for 20 min at room temperature. Images of undifferentiated or differentiated cells were acquired at 20× magnification.

### 4.3. Confocal Imaging

Confocal microscopy was performed for the detection of the hMSCs on the microparticles by means of a Zeiss LSM 5 Pascal Model Confocal Microscope (Carl Zeiss International, Oberkochen, Germany). Ten ILCSs were recovered and stained with phalloidin-FITC, incubated for 1 h at room temperature (Sigma-Aldrich), and with Hoechst 33,258 dye (Sigma-Aldrich) for nuclear staining. Subsequently, ILCSs were seeded on microscope slides.

### 4.4. Release of Gd-NPs In Vitro

To evaluate the release of Gd-NPs in vitro, eight mg of microparticles were embedded into 1% agar contained in 0.5 mL Eppendorf tube. The pH was kept at 7.4 (HEPES buffer, Sigma, St. Louis, MO, USA). The volume of gelified agar was 150 μL. Twenty μL of HEPES buffer were placed on top of the agar to keep the agar well hydrated. MR images were acquired with a Bruker Avance microimaging MR scanner operating at 300 MHz (7 T). T_1_ weighted images were acquired with a mic_msme (multislice-multiecho) pulse sequence, with TR 200 ms, TE 3.6 ms, FOV = 3.5 × 3.5 cm, matrix 128 × 128, 15 slices 0.5 mm thick. T_2_ weighted images were acquired with a mic_RARE pulse sequence, with TR 4000 ms, TE 4.4 ms, FOV = 3.5 × 3.5 cm, matrix 256 × 256, 15 slices 0.5 mm thick. MRI images were acquired up to day 24.

### 4.5. Animal Studies

Animal studies were conducted in accordance with National Institute of Health Guidelines for the Care and Use of Laboratory Animals. All procedures were approved by the Ethics Committee of the University of Turin and the Italian Health Ministry (authorization number Prot. CC652.43). Mice were kept in our institutional animal facility under controlled conditions of temperature, humidity with access to food and water ad libitum.

#### 4.5.1. Cell Implants

Implants to be grafted in mice as described in Scheme 1 were prepared by seeding hMSCs on scaffolds, as described in Section 4.2.1. The cell-loaded scaffolds (or control cell-free scaffolds) were recovered and mixed with 150 μL of MatrigelTM (Becton Dickinson, Franklin Lakes, NJ, USA) and the mixture kept ice cold. Such a mixture solution was surgically implanted subcutaneously on the back of either immunocompromised NSG mice (*n* = 2), or immunocompetent FVB mice (*n* = 2). In detail, mice were anesthetized with i.m. injection of 20 mg kg^−1^ tiletamine–zolazepam (Zoletil 100; Virbac, Milan, Italy) mixed with 5 mg kg^−1^ xylazine (Rompun; Bayer, Milan, Italy) and, under sterile conditions, a small skin incision was made on the back; the mixture was implanted using a large needle. Each mouse received two subcutaneous implants, one implant contained scaffolded hMSCs (+hMSC, placed on the left flank), and the other implant contained the cell-free scaffolds only (−hMSC, right flank). Follow-up by MRI started at Day 0, i.e., the day after surgery. The animals were followed up for 18 days by MRI and sacrificed at Day 20. The implants were excised and subjected to histology.

#### 4.5.2. Magnetic Resonance Imaging

Prior to the acquisition of MR images, mice were anesthetized by i.m. administration of Zoletil and Rompun (see above). MR images were acquired with a Bruker Avance microimaging MR scanner (Bruker BioSpin, Rheinstetten, Germany) operating at 300 MHz (7 T). T_1_ weighted images were acquired with a mic_msme pulse sequence, with TR 200 ms, TE 4.7 ms, FOV = 3.5 × 3.5 cm, matrix 256 × 256, 13 slices 1 mm thick. T_2_ weighted images were acquired with a mic_RARE pulse sequence, with TR 2600 ms, TE 36 ms, FOV = 3.5 × 3.5 cm, matrix 384 × 384, 13 slices 1 mm thick. T_1_w and T_2_w MRI images were acquired at day 0, 5, 7, 10, 12 and 18 post implantation. Image reconstruction was performed with Paravision 5.1 and image analysis by ImageJ 1.47v.

T_2_w MR images at each time point were used to identify the regions of interest (ROI) within matrigel that contained ILCSs. The Signal Enhancement (SE%) was then calculated on the corresponding ROI on co-registered T_1_w MR images according to the formula:

SE% = 100 × (SI_ROI_ − SI_ref_)/SI_ref_ (1)

where SI_ROI_ is the signal intensity measured in the ROI drawn around the ILCSs, and SI_ref_ is the signal intensity measured on a reference sample. The reference sample was a vial containing phosphate buffered saline, placed next to the imaged animal. As mice were imaged at different times, it was not always possible to take and compare slices exactly at the same position. To have a consistent quantitation of the time course of contrast enhancement, a set of 13 axial slices (each one mm thick) were placed such to have a complete coverage of both +hMSC and –hMSC implants (Figure S8). T_2_w and T_1_w MR images were co-registered. T_2_w images were used to define the ROI around ILCS, while T_1_w weighted images were used to measure the gadolinium dependent SE due to released Gd-NPs. The change of SE was averaged on the entire volume of the implant at every time point in the serial images (with exclusion of slices suspected of partial volume artefacts; the smallest implant spanned across four valid slices at least).

#### 4.5.3. Histology

Cell implants were excised from mice at the end of the follow-up period (Day 20), and explants were cut into 5 μm-thick sections. Sections were stained with hematoxylin and eosin (H&E) or Masson trichrome, or Sirius red. Fluorescence confocal microscopy was performed for the detection of the hMSCs in the implant. Sections were labelled with rabbit anti-HLA antibody (Cod, ab52922 Abcam, Cambridge Science Park, UK). Alexa Fluor anti-rabbit antibody (Molecular Probes, Leiden, the Netherlands) was used as secondary antibody. Hoechst dye was added for nuclear staining. Microscopy analysis was performed using an Aptome Zeiss (Carl Zeiss International, Oberkochen, Germany).

## 5. Conclusions

Microparticles endowed with controlled-release of Gd-NPs enable to integrate the cell scaffolding function with an imaging function. Our initial in vivo study indicates that the MRI follow-up of cell-loaded scaffolds can report on the host inflammatory response to the cell graft. However, in its current form, this method does not provide quantitative information about the viability of transplanted cells.

## Supplementary Materials

The following are available online at https://www.mdpi.com/2079-4983/10/3/28/s1, Table S1, and Figures S1–S8. Table S1. List of the antibodies used in this study. Figure S1. Transmission electron micrographs of particle sections, showing electron dense Gd-NPs with diameter of 1–2 nm. Figure S2. Optical images at the inverted microscope, showing hMSCs after 3 days seeding with ILCSs. The arrows show hMSCs on the particle surface (A) or at the junction between particles (B–D). Figure S3. SEM micrographs of ILCSs seeded with hMSCs (after 10 days culture) at (A) 500× and (B) 200× magnification. Cells have been fixed with formalin for SEM. Cells appear mostly located at the junction between adjacent microparticles. Figure S4. Assessment of the multipotentiality of hMSCs after incubation up to 20 days with ILCS. (A) Multipotentiality markers by flow cytometry analysis; (B) Differentiation into adipocytes (middle, Oil Red staining) or osteocytes (right, Alizarin Red staining). The left panel is the control. Figure S5. Expansions of MR images around the −hMSCs grafts (contralateral to the implants shown in Figure 5, main text) in an immunocompromised NSG mouse (a–d) and an immunocompetent FVB mouse (e–h). Similar to +hMSCs implants, activation of contrast enhancement in T_1_w-MR images is observed in the immunocompromised mouse on going from day-0 (b) to day-12 (d). Poor activation of contrast enhancement is observed for the immunocompetent mouse (f,h). Figure S6. Photograph of the Matrigel-based hydrogel embedding cell-loaded ILCSs (pink spots) excised from an immunocompromised mouse 20 days after implantation. Figure S7. Histology of -hMSC subcutaneous cell implants excised from a representative immunocompromised NSG mouse (a–c) and immunocompetent FVB mouse (d–f). (a,d) H&E stains; (b,e) Masson stains; (c,f) Sirius red stains. Arrows indicate microspheres delimited by an intense fibrotic reaction. Arrow-heads are pointing the vascular organization of the matrigel. Double arrows are indicating macrophage foamy cells. Scale bar: 50 μm for a,b,d,e; 25 μm for c,f. Figure S8. Schematics about the geometry of MRI slices across ILCS implants to measure the signal enhancement (see main text, Section 4.5.2.).
