# Supplementary material for: Gadolinium-Labelled Cell Scaffolds to Follow-up Cell Transplantation by Magnetic Resonance Imaging
Source: J Funct Biomater. 2019 Jul 2;10(3):28. doi: 10.3390/jfb10030028 (PMC6787680; doi:10.3390/jfb10030028)
Supplement: Supplementary file 1 [file jfb-10-00028-s001.pdf]

Journal of *Functional Biomaterials*

SUPPORTING INFORMATION

Gadolinium-labelled cell scaffolds to follow-up cell transplantation by MRI

Valeria Catanzaro,[1] Giuseppe Digilio,[1,*] Federico Capuana,[2] Sergio Padovan,[3] Juan C. Cutrin,[2] Fabio Carniato,[1] Stefano Porta,[2] Cristina Grange,[4] Nenad Filipović,[5] and Magdalena Stevanović[5]

1 *Department of Science and Technologic Innovation, Università del Piemonte Orientale "Amedeo Avogadro", Viale T. Michel 11, I-15121 Alessandria, Italy.*

2 *Department of Molecular Biotechnology and Health Science & Center for Molecular Imaging, University of Turin, Via Nizza 52, 10126 Torino, Italy.*

3 *Institute for Biostructures and Bioimages (CNR) c/o Molecular Biotechnology Center Via Nizza 52, 10126 Torino, Italy.*

4 *Department of Medical Sciences, University of Turin, Via Nizza 52, 10126 Torino, Italy.*

5 *Institute of Technical Sciences of the Serbian Academy of Sciences and Arts, Knez Mihailova 35/IV, 11000 Belgrade, Serbia.*

Supplementary Table

Table S1. List of the antibodies used in this study

Antigen	Company	Code	Dilution
HLA	Abcam	Cod: ab52922	1:100
CD146	Miltenyi Biotec	Cod: 130-092-851	1:100
CD105	Miltenyi Biotec	Cod: 130-098-774	1:100
CD90	Miltenyi Biotec	Cod: 130-095-403	1:100
CD73	Miltenyi Biotec	Cod: 130-095-182	1:100
CD44	Miltenyi Biotec	Cod: 130-095-180	1:100
Alpha 5 Integrin	BD Pharmingen	Cod: 5555617	1:100
CD14	BD Pharmingen	Cod: 555397	1:100
CD34	BD Pharmingen	Cod: 555821	1:100
CD45	BD Pharmingen	Cod: 555482	1:100
Phalloidin FITC	Sigma	P5282	1:100

Supplementary Figures

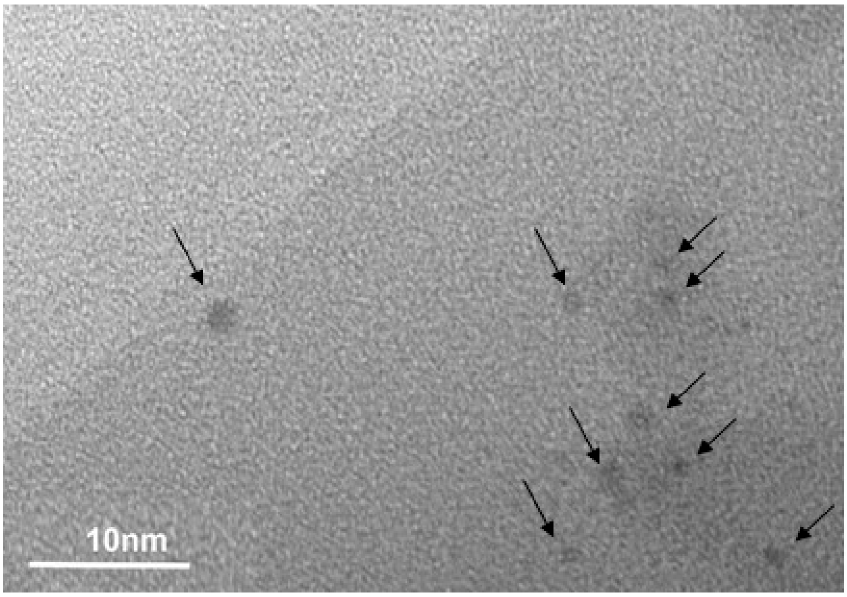

Figure S1 Transmission electron micrographs of particle sections, showing electron dense Gd-NPs with diameter of 1-2 nm.

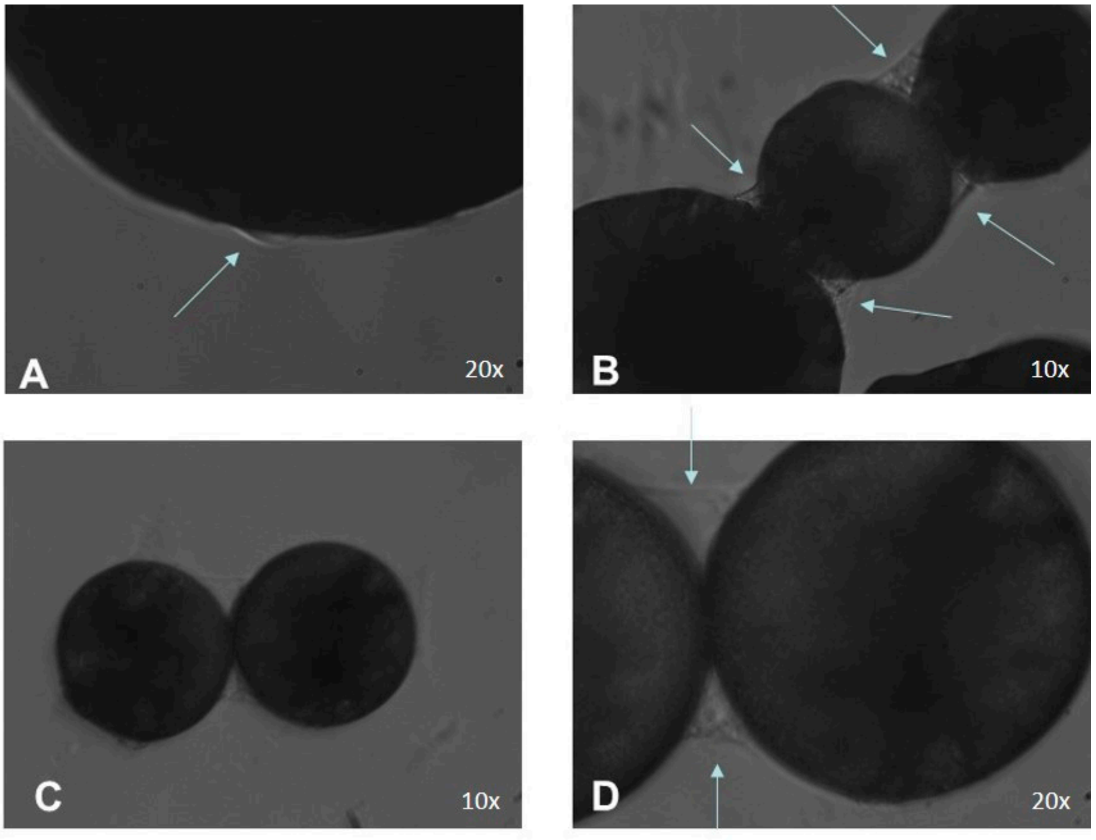

Figure S2 Optical images at the inverted microscope, showing hMSCs after 3 days seeding with ILCSs. The arrows show hMSCs on the particle surface (**A**) or at the junction between particles (**B**, **C**, **D**).

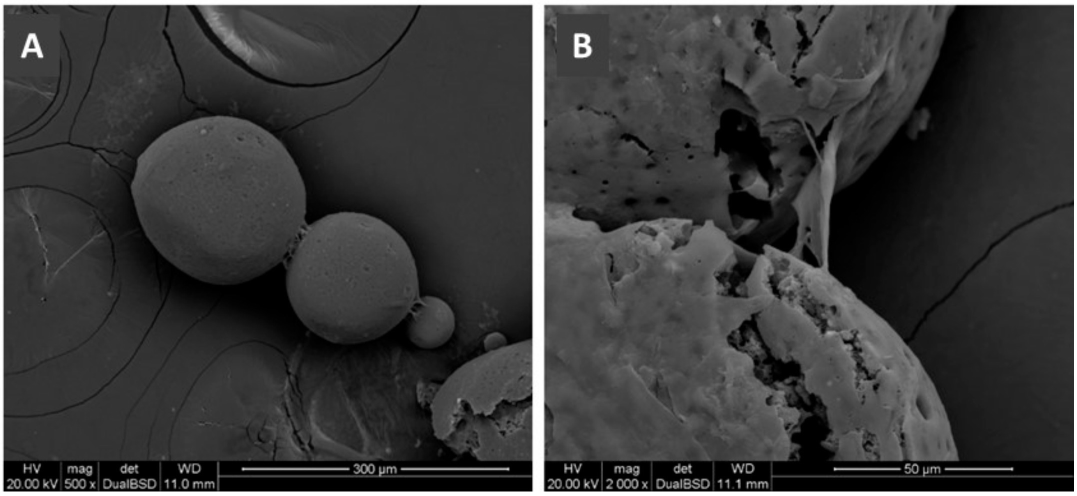

Figure S3 SEM micrographs of ILCSs seeded with hMSCs (after 10 days culture) at ***(A)*** 500x and ***(B)*** 200x magnification. Cells have been fixed with formalin for SEM. Cells appear mostly located at the junction between adjacent microparticles.

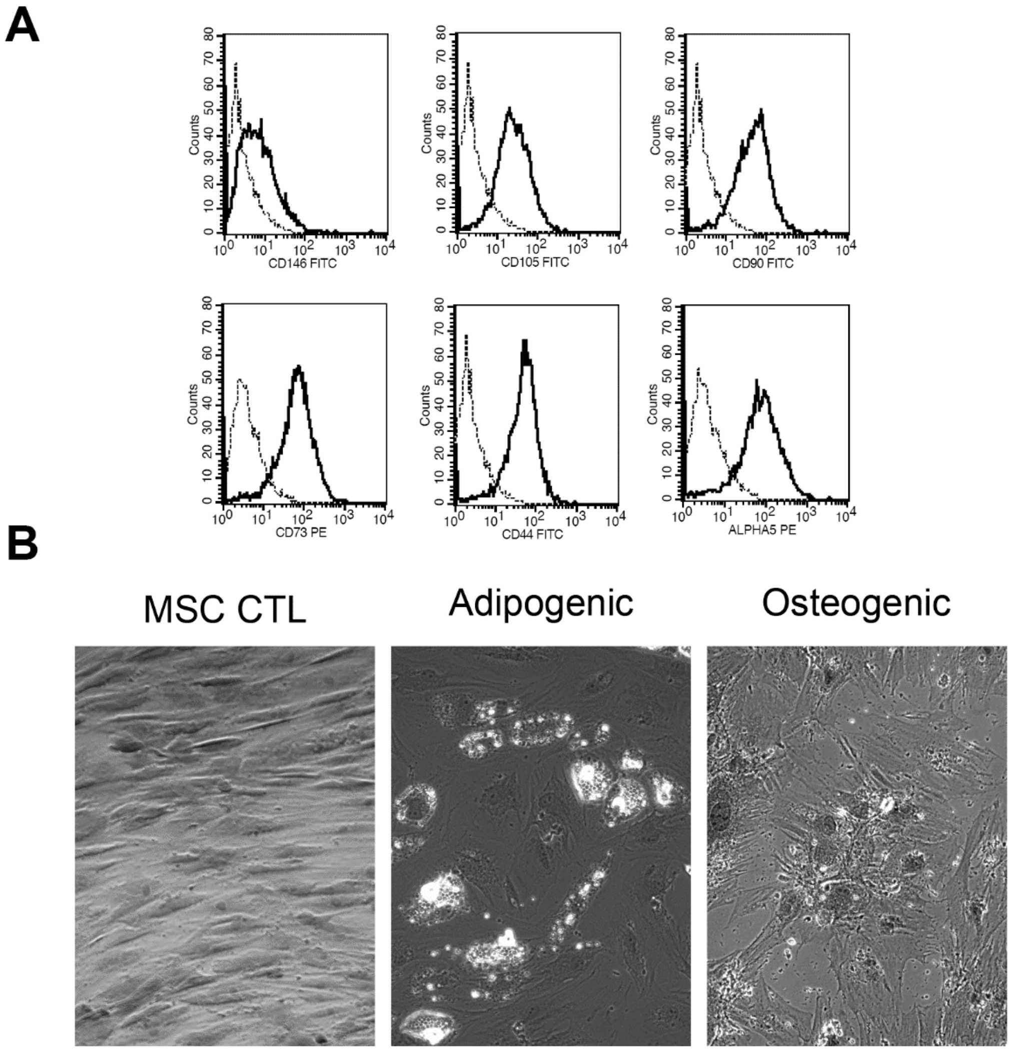

Figure S4 Assessment of the multipotentiality of hMSCs after incubation up to 20 days with ILCS. ***A)*** Multipotentiality markers by flow cytometry analysis; ***B)*** Differentiation into adipocytes (middle, Oil Red staining) or osteocytes (right, Alizarin Red staining). The left panel is the control.

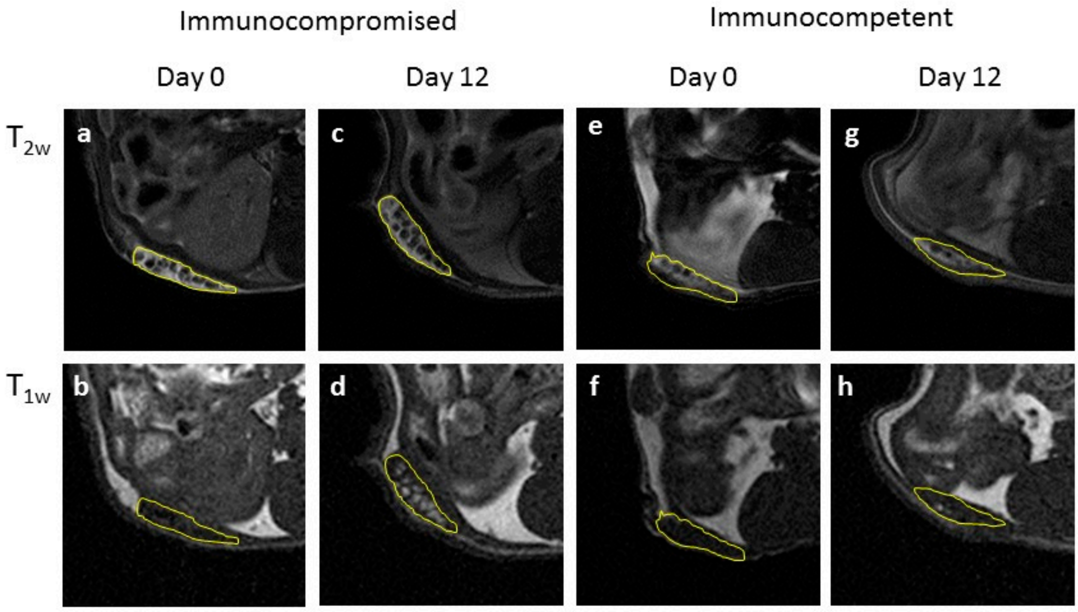

Figure S5 Expansions of MR images around the − hMSCs grafts (contralateral to the implants shown in Fig. 5, main text) in an immunocompromised NSG mouse ***(a-d)*** and an immunocompetent FVB mouse ***(e-h)***. Similar to +hMSCs implants, activation of contrast enhancement in T_1w-MR images is observed in the immunocompromised mouse on going from day-0 (***b***) to day-12 (***d***). Poor activation of contrast enhancement is observed for the immunocompetent mouse (***f,h***).

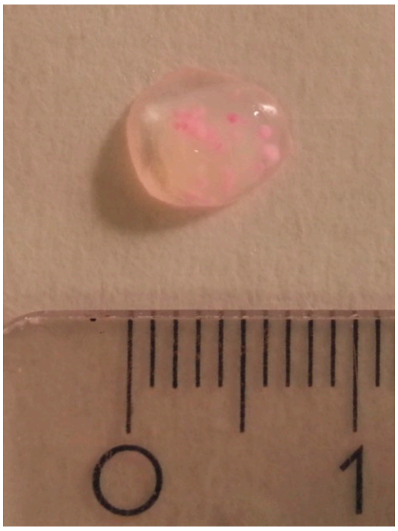

Figure S6 Photograph of the Matrigel-based hydrogel embedding cell-loaded ILCSs (pink spots) excised from an immunocompromised mouse 20 days after implantation.

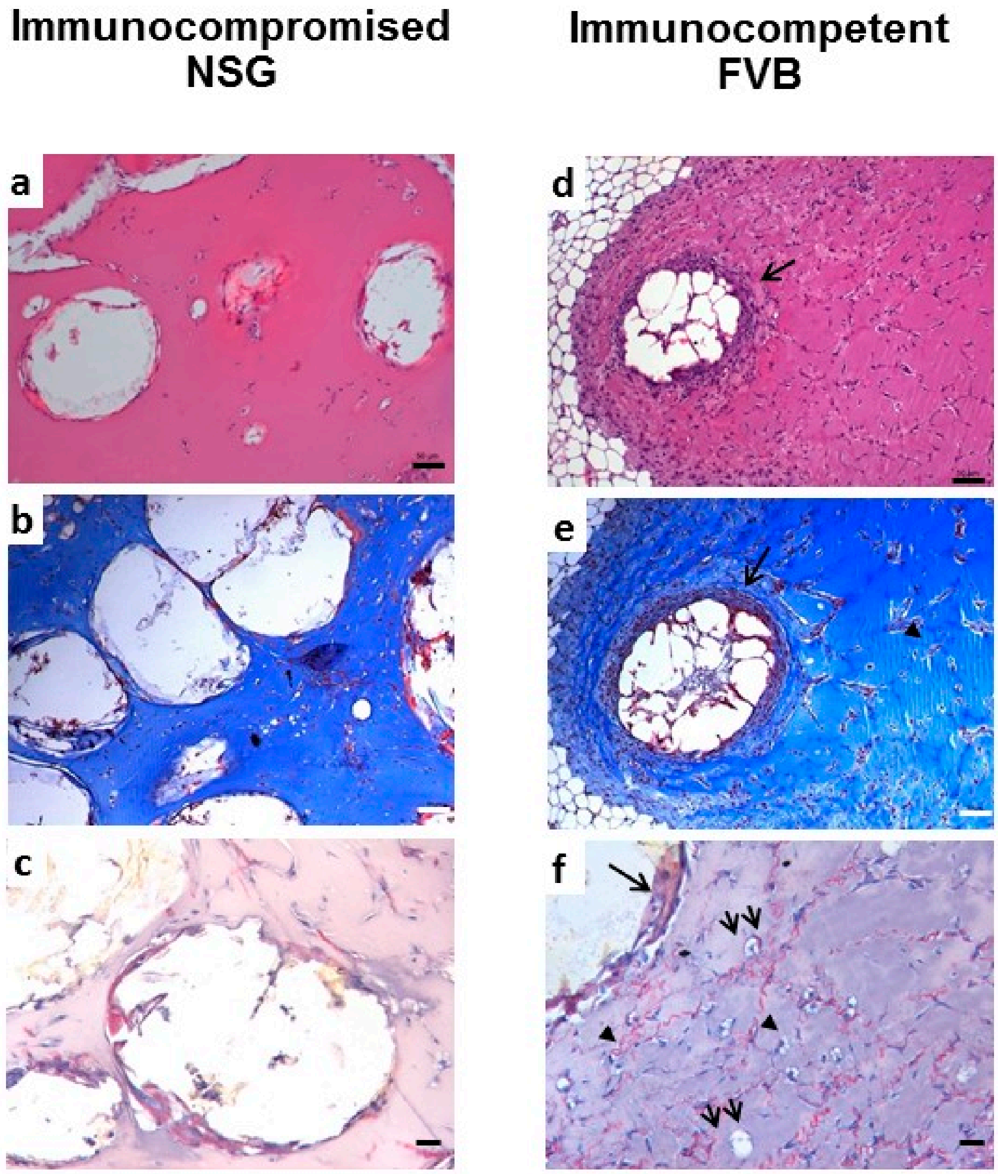

Figure S7 Histology of -hMSC subcutaneous cell implants excised from a representative immunocompromised NSG mouse (**a-c**) and immunocompetent FVB mouse (**d-f**). (**a,d**) H&E stains; (**b,e**) Masson stains; (**c,f**) Sirius red stains. Arrows indicate microspheres delimited by an intense fibrotic reaction. Arrow-heads are pointing the vascular organization of the matrigel. Double arrows are indicating macrophage foamy cells. Scale bar: 50 µm for **a**,**b**,**d**,**e**; 25 µm for **c**,**f** .

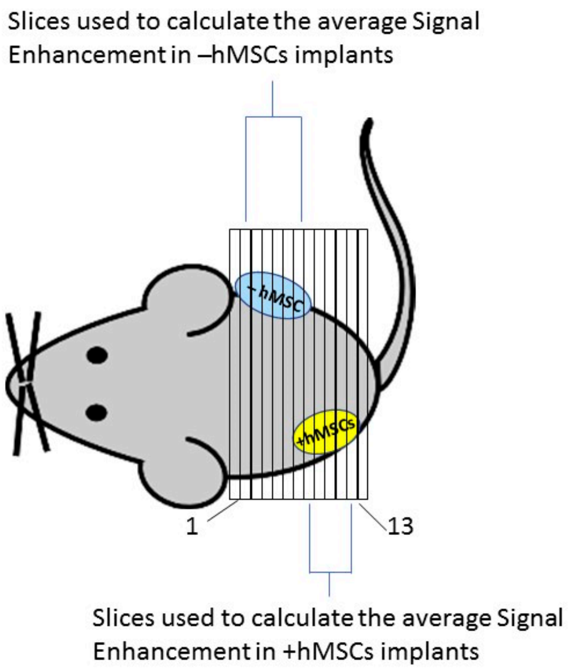

Figure S8 Schematics about the geometry of MRI slices across ILCS implants to measure the signal enhancement (see main text, Section 4.5.2.).
